# Cholecystokinin Receptor Antagonist Suppresses Melanoma Growth by Inducing Apoptosis of Tumor Cells

**DOI:** 10.1016/j.xjidi.2022.100153

**Published:** 2022-09-02

**Authors:** Atsuko Funakoshi, Tetsuya Honda, Taisuke Ito, Yoshiki Tokura

**Affiliations:** 1 Department of Dermatology, Hamamatsu University School of Medicine, Hamamatsu, Japan; 2 Department of Dermatology & Skin Oncology, Chutoen General Medical Center, Kakegawa, Japan; 3 Allergic Disease Research Center, Chutoen General Medical Center, Kakegawa, Japan

**Keywords:** 7AAD, 7-amino-actinomycin D, CCK, cholecystokinin, CCKAR, cholecystokinin receptor A, CCKBR, cholecystokinin receptor B, CFSE, carboxyfluorescein diacetate succinimidyl ester, KC, keratinocyte, SCC, squamous cell carcinoma

## Abstract

Melanoma is a malignant skin tumor with high metastatic activity. Although melanoma has been well-studied, its cellular kinetics remain elusive. The cholecystokinin (CCK) receptor is expressed in various types of tumors because CCK promotes the survival and proliferation of tumor cells. Thus, we hypothesized that the growth of melanoma was positively regulated by signals from the CCK receptor and sought to investigate whether CCK receptor antagonists affect the growth of melanoma cells expressing CCK receptor. Immunohistochemically, the CCK receptor A is expressed in the clinical specimens of melanoma. CCK receptor antagonists decreased the viability of melanoma cells by suppressing cell division and promoting apoptosis. CCK receptor antagonists also decreased the mitochondrial membrane potential through enhanced gene expression of the proapoptotic protein, *Bcl2*-associated X, and tumor suppressor, *p53*, suggesting that the antagonist induced the apoptosis of melanoma cells in a mitochondria-dependent manner. In addition, a caspase 3 inhibitor, Z-DEVD-FMK, partially blocked the antiviability of the antagonist, indicating that caspase 3 is involved in antagonist-induced apoptosis. Notably, tumor growth was attenuated when a CCK receptor antagonist was locally administered to the melanoma-bearing mice. Therefore, our study suggests the therapeutic potential of CCK receptor antagonists in the treatment of skin cancer.

## Introduction

Melanoma is a malignant skin tumor with high metastatic activity and is one of the main causes of cancer-related deaths. Recently, large-scale genomic profiling identified considerable heterogeneity in melanoma. Characterization of somatic driver sequence variants can be used to distinguish between different subtypes of melanoma, such as nonacral cutaneous melanoma, desmoplastic melanoma, acral melanoma, mucosal melanoma, and uveal melanoma, leading to the development of many targeted therapies against tumors (Guhan et al., 2021). Although targeted therapies exist for certain sequence variants, such as *BRAF* and *KIT*, other genotypes respond to newer-generation immune therapies. Immune checkpoint inhibitors have revolutionized the management of advanced melanoma (Carlino et al., 2021). PD-L1 expression is a reliable prognostic marker for melanoma (Hino et al., 2010).

Further studies on the cellular kinetics of melanoma can aid in the development of new treatment options. Apoptosis is a well-orchestrated system involving a large number of regulatory molecules that eliminate tumor cells through the self-immune system or chemotherapeutic agents. Thus, the molecular mechanisms underlying the apoptosis of tumor cells have been extensively studied to develop new therapeutic strategies for malignant neoplasms, such as melanoma. The molecules that regulate apoptosis in melanoma include the tumor suppressor p53, APAF1, Noxa, p53-upregulated modulator of apoptosis, BCL-2 family proteins (BCL-2, BAX, and BAK), and caspases (Hussein et al., 2003). These molecules have been proposed as potential prognostic markers or therapeutic targets in melanoma (Fecker et al., 2006; Lavrik et al., 2005; Lu et al., 2014).

Cholecystokinin (CCK) is expressed in the gastrointestinal system and CNS, where it functions as a peptide hormone (Zeng et al., 2020). Under physiological conditions, CCK exerts various regulatory effects on digestive hormone secretion from the pancreas, gallbladder contraction, and satiety in the brain (Zeng et al., 2020). CCK transduces signals through two types of receptors, the CCK receptor A (CCKAR) and CCK receptor B (CCKBR), which are G protein‒coupled receptors (Dufresne et al., 2006). CCK receptors are expressed not only in normal tissues but also in various types of cancer, such as pancreatic, colon, and lung cancers (Dufresne et al., 2006; Smith et al., 2016). CCK promotes the proliferation and survival of tumor cells, and thus the blockade of the CCK receptor with its antagonists suppresses the proliferation and induces the apoptosis of tumor cells, leading to the inhibition of tumor growth in vivo (Morimoto et al., 1993; Ponnusamy et al., 2016; Smith et al., 2016). Mathieu et al. (2005) reported that selective CCKBR antagonists delayed the growth of C32 human melanoma xenografts, which may be attributed to their inhibitory effect on neoangiogenesis. Because C32 melanoma cells neither expressed CCKBR nor CCKAR, it is unlikely that CCKBR antagonists exerted their antitumor effects on C32 melanoma xenografts through direct effects on melanoma cells (Mathieu et al., 2005). However, it remains to be clarified whether the CCK/CCK receptor signaling directly affects the growth of other melanoma cell lines.

We have previously shown that CCK is abundantly expressed in human and mouse epidermal keratinocytes (KCs) in a steady state, and its expression is attenuated by IL-17A in psoriatic skin lesions as well as an imiquimod-induced psoriasis model (Funakoshi et al., 2019). CCK plays an anti-inflammatory role by suppressing cytokine production from epidermal KCs through CCKAR (Funakoshi et al., 2019). IL-17A decreases the expression levels of both CCK and CCKAR in KCs (Funakoshi et al., 2020, 2019). We also showed that CCK suppresses substance P‒induced itch by suppressing mast cell degranulation (Fukamachi et al., 2011). These findings indicate that CCK plays a significant role in both the physiological and pathological conditions of the skin. However, it remains unknown whether CCK and its receptors are involved in the progression of various types of skin cancer, such as melanoma.

In this study, we showed the role of CCK receptor signals in the proliferation and survival of melanoma and squamous cell carcinoma (SCC) cells and presented a therapeutic strategy for skin tumors on the basis of the antitumor properties of CCK receptor antagonists. In vitro experiments using several skin tumor cell lines showed that CCK receptor antagonists suppress the proliferation and survival of melanoma and SCC cells. Topical administration of a CCK receptor antagonist attenuated the growth of melanoma and SCC cells in allograft and xenograft models, respectively. These results suggest that CCK and CCK receptors are promising targets for the treatment of melanoma and other skin tumors.

## Results

### CCKAR is expressed in human melanoma cells and melanoma and SCC cell lines

**Figure 1:**
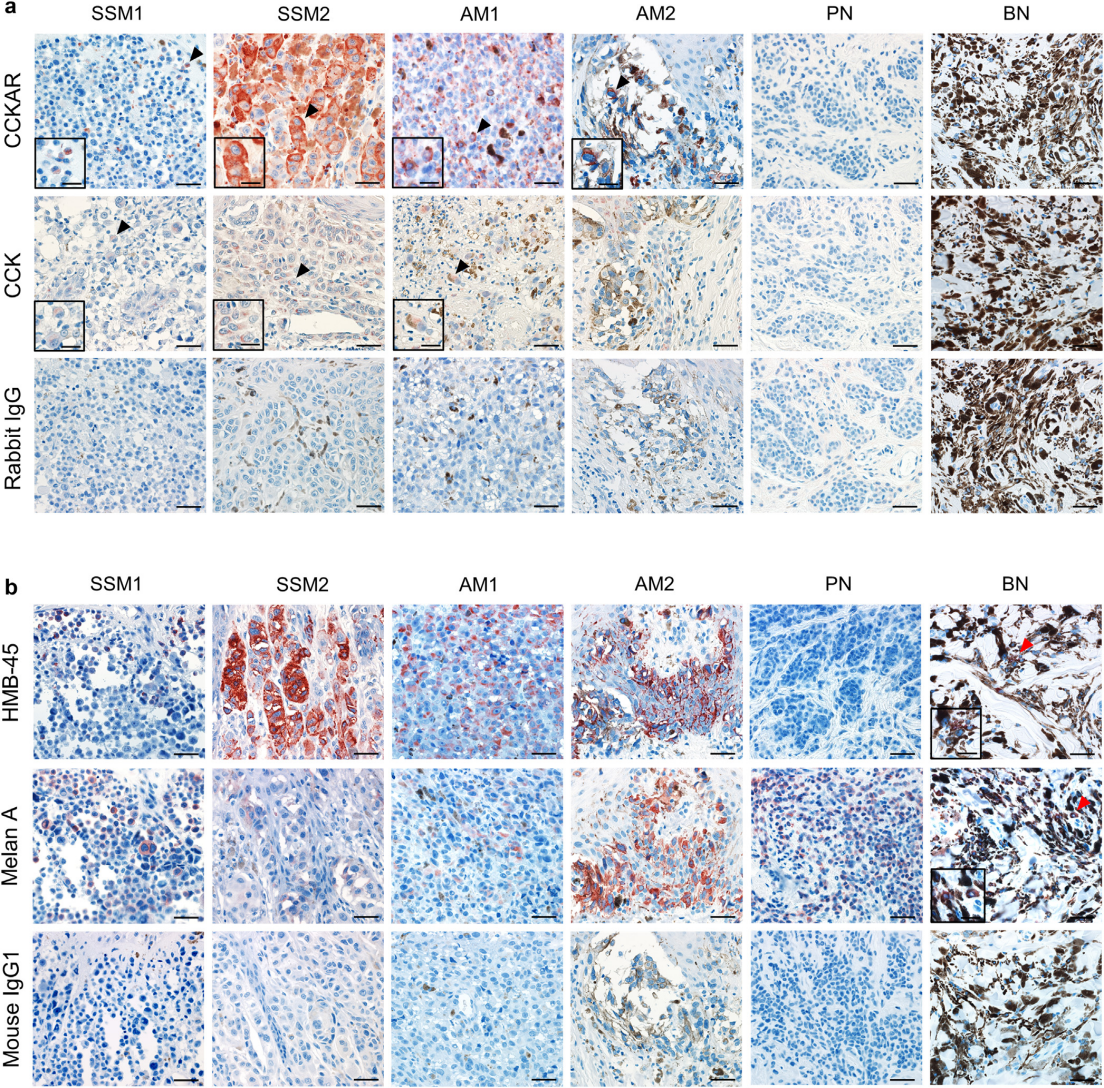
**CCKAR is expressed in melanoma cells.** (**a, b**) Immunohistochemical images of SSM, AM, PN, and BN stained with the anti-CCKAR, anti-CCK, rabbit IgG isotype control, anti‒HMB-45, anti‒Melan A, and mouse IgG1 isotype control mAb. Positive staining (red) for CCKAR and CCK in tumor cells indicated by black arrowheads is shown in the inserts at high magnification. Red arrowheads indicate positive staining with HMB-45 and Melan A in BN, as shown in the inserts at high magnification. The images are representative of five SSM, five AM, four PN, and three BN cases. Bar = 40 and 20 μm for the inserts. AM, acral melanoma; BN, blue nevus; CCK, cholecystokinin; CCKAR, CCK receptor A; PN, pigmented nevus; SSM, superficial spreading melanoma.

**Figure 2:**
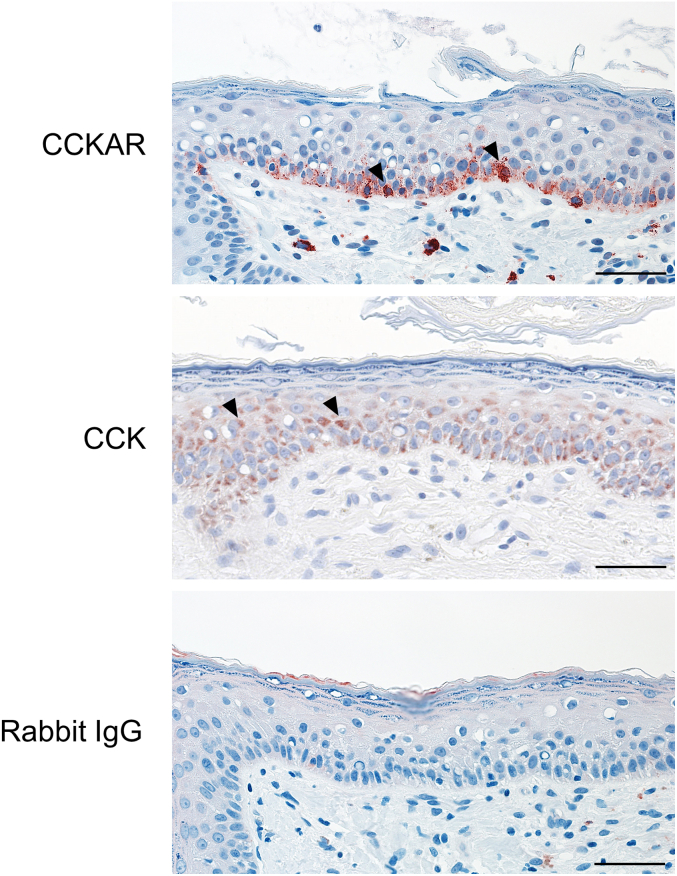
**Immunohistochemical images of normal human skin.** Normal human skin specimens were stained with anti-CCKAR, anti-CCK, or rabbit IgG isotype control antibodies. Arrowheads indicate positive staining (red) for CCKAR and CCK in epidermal keratinocytes. Images are representative of three individuals. Bar = 40 μm. CCK, cholecystokinin; CCKAR, cholecystokinin A receptor.

Some skin constituents bear CCKAR and its ligand, CCK, because their expression is observed in epidermal KCs (Funakoshi et al., 2019). First, we determined the expression levels of CCKAR and CCK in melanoma skin specimens. CCKAR was immunohistochemically stained for superficial spreading melanoma (n = 5) and acral melanoma (n = 5) skin lesions. We found that CCKAR was positively stained in the cytoplasm of tumor cells in all superficial spreading melanoma and acral melanoma cases, as represented by two cases of each type (Figure 1a, upper panel). CCK was detected in three of five cases of superficial spreading melanoma and two of five cases of acral melanoma (Figure 1a, middle panel). Positive staining was not observed in the specimens stained with rabbit IgG isotype control antibodies, although some melanin-containing cells were observed (Figure 1a, lower panel). We confirmed that CCKAR^+^ and CCK^+^ cells were melanoma cells by staining these specimens with antibodies against the common markers of melanoma, HMB-45 and Melan A (Figure 1b) (Ohsie et al., 2008). In addition, we examined the expression levels of CCKAR and CCK in pigmented nevus (n = 4; HMB-45 negative and Melan A positive) and blue nevus (n = 3; HMB-45-weakly positive and Melan A positive) and found no positively stained cells in all cases of pigmented nevus and blue nevus, as represented by one case of each nevus (Figure 1a). The specificity of the antibodies was also confirmed by staining normal human skin cells with anti-CCKAR, anti-CCK, or rabbit IgG isotype control antibodies (Figure 2). Similar to our previous studies (Funakoshi et al., 2020, 2019), epidermal KCs were positively stained with anti-CCKAR and anti-CCK antibodies but not with rabbit IgG isotype control antibody.

**Figure 3:**
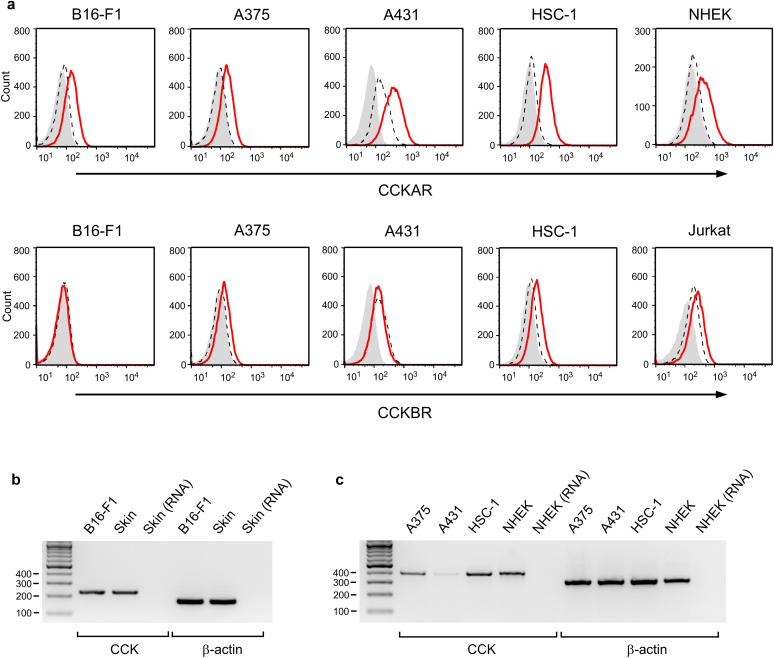
**Expression levels of CCK receptors and CCK in melanoma and SCC cells.** (**a**) Flow cytometric analysis of CCK receptors in melanoma and SCC cells. B16-F1, A375, A431, and HSC-1 cells were stained with the anti-CCKAR antibody (red line, upper panel), anti-CCKBR antibody (red line, lower panel), isotype control antibody (black dashed line), or second antibody only (filled gray). NHEKs and Jurkat cells were used as positive controls for CCKAR and CCKBR, respectively. (**b, c**) RT-PCR analysis of mRNA for mouse CCK and β-actin and (**b**) for human CCK and (**c**) β-actin. Product length was as follows: 224 bp for mouse CCK, 163 bp for mouse β-actin, 389 bp for human CCK, and 305 bp for human β-actin. Data are representative of two independent experiments. CCK, cholecystokinin; CCKAR, cholecystokinin A receptor; CCKBR, cholecystokinin B receptor; NHEK, normal human epidermal keratinocyte; SCC, squamous cell carcinoma.

Next, we evaluated the expression levels of CCK and its receptors in several skin tumor cell lines. We used normal human epidermal KCs and Jurkat cells as positive controls for CCKAR and CCKBR, respectively (Funakoshi et al., 2020, 2019; Lignon et al., 1991). Flow cytometric analysis revealed that B16-F1 mouse melanoma and A375 human melanoma cells expressed CCKAR, whereas CCKBR was hardly detected in these cells (Figure 3a). A431 and HSC-1 human SCC cells also showed significantly high CCKAR expression levels (Figure 3a, upper panel). CCKBR was detected in HSC-1 cells at a level comparable with that in Jurkat cells (Figure 3a, lower panel) (Lignon et al., 1991); however, it was not detected in A431 cells. We then evaluated the expression levels of *CCK* mRNA through RT-PCR and found that *CCK* mRNA was detected in all melanoma and SCC cells (Figure 3b and c). As we reported previously (Funakoshi et al., 2019), mouse skin and normal human epidermal KCs expressed *CCK* mRNA, whereas the *CCK* gene was not amplified when RNA was used as a template for RT-PCR. We determined the nucleotide sequences of RT-PCR products through direct sequencing methods and confirmed that the nucleotide sequences were identical to those of CCK (mouse CCK, NM_031161; human CCK, NM_000729; data not shown).

**Figure 4:**
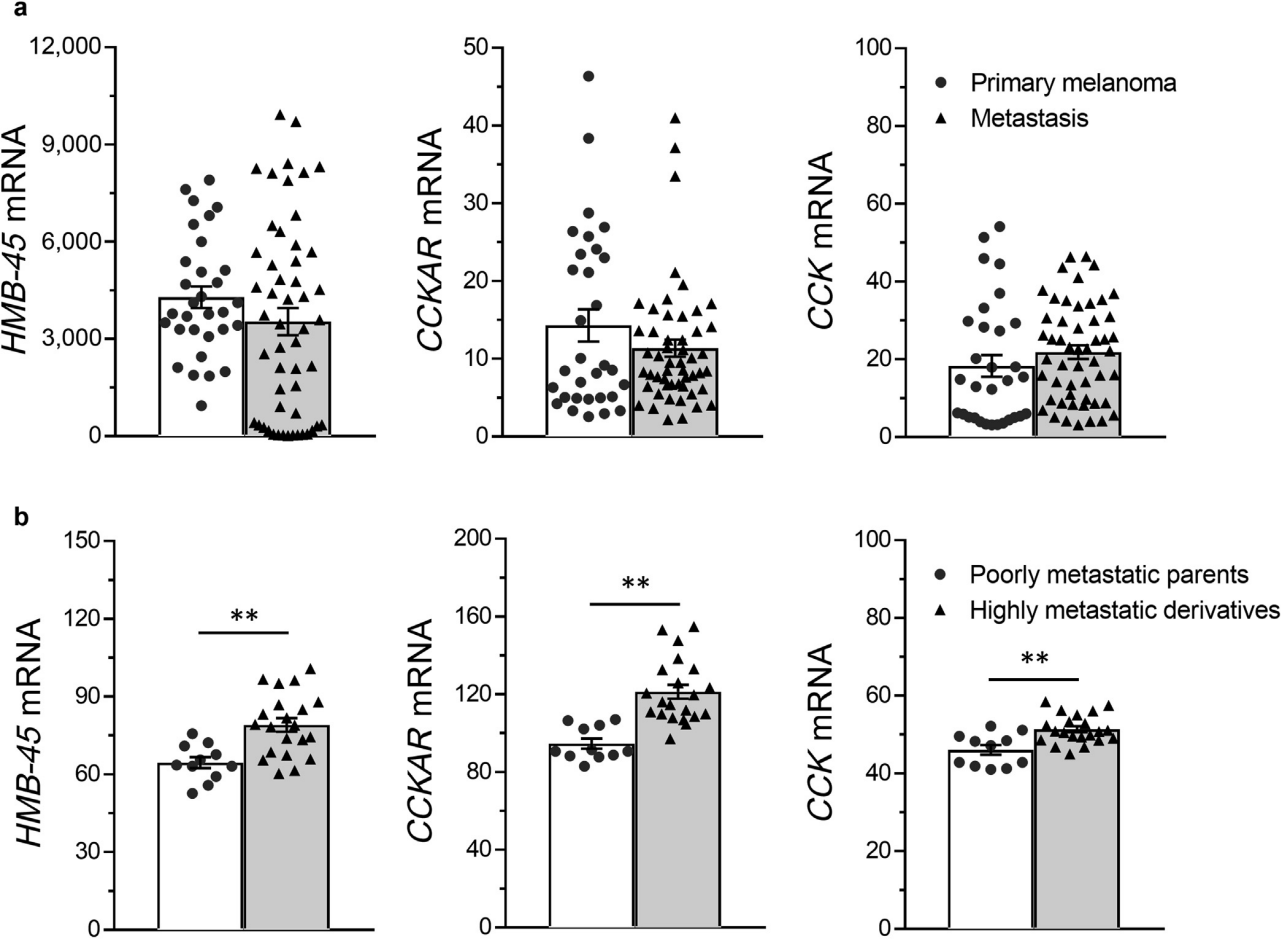
**Expression levels of HMB-45, CCKAR, and CCK in clinical melanoma and A375 human melanoma cells.** Data of (**a**) clinical melanoma and (**b**) A375 cells were obtained from GSE8401 and GSE7929, respectively. (**b**) Poorly metastatic A375 parents were intravenously injected into nude mice, and highly metastatic derivatives were obtained from the lung. The data were expressed as the mean ± SEM. *P*-values were calculated by two-tailed *t*-test. ∗∗*P* < 0.01. CCK, cholecystokinin; CCKAR, cholecystokinin A receptor;

We analyzed two published gene expression profiles for human clinical melanoma (GSE8401) and A375 melanoma (GSE7929) cells (Xu et al., 2008). As shown in Figure 4a and b, HMB-45‒expressing clinical melanoma and A375 melanoma cells coexpressed genes for CCKAR and CCK. Moreover, in the A375 dataset, the expression levels of CCKAR and CCK in highly metastatic derivatives were significantly higher than those in poorly metastatic parent A375 cells.

### CCK receptor antagonists inhibit the viability and proliferation of melanoma and SCC cells

In vivo and in vitro studies have revealed that the CCK/CCK receptor engagement is involved in the proliferation and survival of several tumor cells (Morimoto et al., 1993; Ponnusamy et al., 2016; Smith et al., 2016). Thus, it is hypothesized that the growth of melanoma and SCC is also regulated by CCKAR signaling. The simultaneous expression of CCK and CCK receptors implies that the CCK receptor‒mediated signaling operates in an autocrine or paracrine manner as a growth mechanism in these skin tumors. Several in vitro studies using various types of tumor cells have shown that CCK receptor agonists function as GFs, and hence, the blockade of CCK receptors inhibits their proliferation and survival (Oikonomou et al., 2008; Smith et al., 2016).

**Figure 5:**
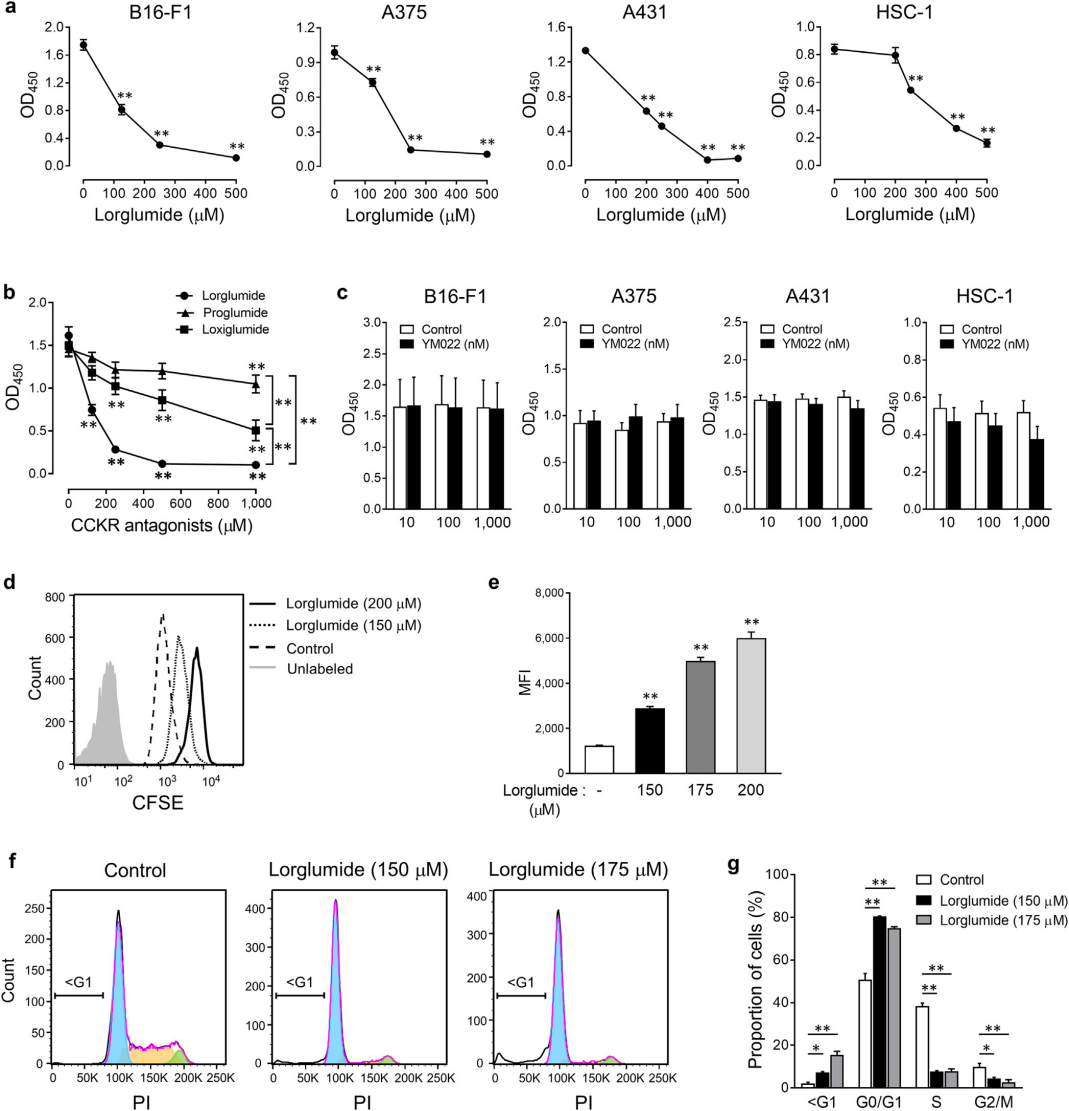
**CCK receptor antagonists reduce the viability and proliferation of skin cancer cells.** (**a**) B16-F1, A375, A431, and HSC-1 cells were treated with the indicated concentrations of lorglumide for 48 hours, and cell viability was determined using a WST-8 assay. ∗∗*P* < 0.01 versus control. (**b**) WST-8 assay of B16-F1 melanoma cells treated with the indicated concentrations of lorglumide, proglumide, or loxiglumide for 48 hours. ∗∗*P* < 0.01. (**c**) WST-8 assay of B16-F1, A375, A431, and HSC-1 cells treated with YM022 or DMSO (control) for 48 hours. (**a**–**c**) Data are expressed as the mean ± SEM of three independent experiments performed in duplicate. B16-F1 melanoma cells were labeled with CFSE and treated with lorglumide for 48 hours. Representative histogram of (**d**) CFSE and (**e**) MFI presented as the mean ± SEM of three independent experiments performed in duplicate. ∗∗*P* < 0.01 versus control. (**f, g**) Cell cycle analysis was performed using flow cytometry after B16-F1 cells were treated with lorglumide for 48 hours. Representative histogram of (**f**) PI and (**g**) percentages of cells distributed in G0/G1, S, G2/M, and sub-G1 phases. ∗*P* < 0.05 and ∗∗*P* < 0.01. (**g**) Data are expressed as the mean ± SEM of three independent experiments. *P*-values were calculated using (**a, e, g**) one-way or (**b**) two-way ANOVA with Bonferroni’s adhoc test for multiple pairwise comparisons. CCK, cholecystokinin; CFSE, carboxyfluorescein diacetate succinimidyl ester; MFI, mean fluorescence intensity; OD, optical density; PI, propidium iodide; WST, water-soluble tetrazolium salt.

We examined the effects of CCK receptor antagonists on the viability of skin tumor cells. We treated B16-F1, A375, A431, and HSC-1 cells with lorglumide, a selective CCKAR antagonist, for 48 hours, and cell viability was determined using the water-soluble tetrazolium salt-8 assay. Lorglumide significantly decreased the viability of melanoma and SCC cells in a dose-dependent manner (Figure 5a). The antiviability properties of other CCK receptor antagonists were evaluated in B16-F1 cells (Figure 5b). The viability of B16-F1 cells was also decreased by treatment with loxiglumide, another selective CCKAR antagonist, and proglumide, a nonselective CCKAR/CCKBR antagonist. Among the three antagonists, lorglumide was found to have the most potent antiviability capacity. Although HSC-1 cells expressed CCKBR, the CCKBR antagonist, YM022, did not affect the viability of either melanoma or SCC cells even at a dose 100 times that of the half-maximal inhibitory concentration (Figure 5c), suggesting that the CCK receptor antagonist decreases the viability of melanoma and SCC cells through CCKAR rather than through CCKBR.

To assess whether the antiviability of the CCK receptor antagonist is associated with its suppressive effect on cell division, B16-F1 cells were labeled with carboxyfluorescein diacetate succinimidyl ester (CFSE) and treated with lorglumide. As shown in Figure 5d, the fluorescence intensity of CFSE in lorglumide-treated cells was higher than that in the untreated control cells, indicating that lorglumide suppressed cell division in B16-F1 cells. Lorglumide inhibited cell division in a dose-dependent manner, as assessed by the mean fluorescence intensity of CFSE (Figure 5e). To confirm the suppressive effect of lorglumide on cell division, we performed a cell cycle analysis of B16-F1 cells treated with lorglumide. As shown in Figure 5f and g, lorglumide significantly increased the percentage of cells in the G0/G1 phase while decreasing the percentage of cells in the S and G2/M phases, indicating that lorglumide induced G0/G1 arrest in B16-F1 cells.

### CCK receptor antagonist induces the apoptosis of melanoma cells

**Figure 6:**
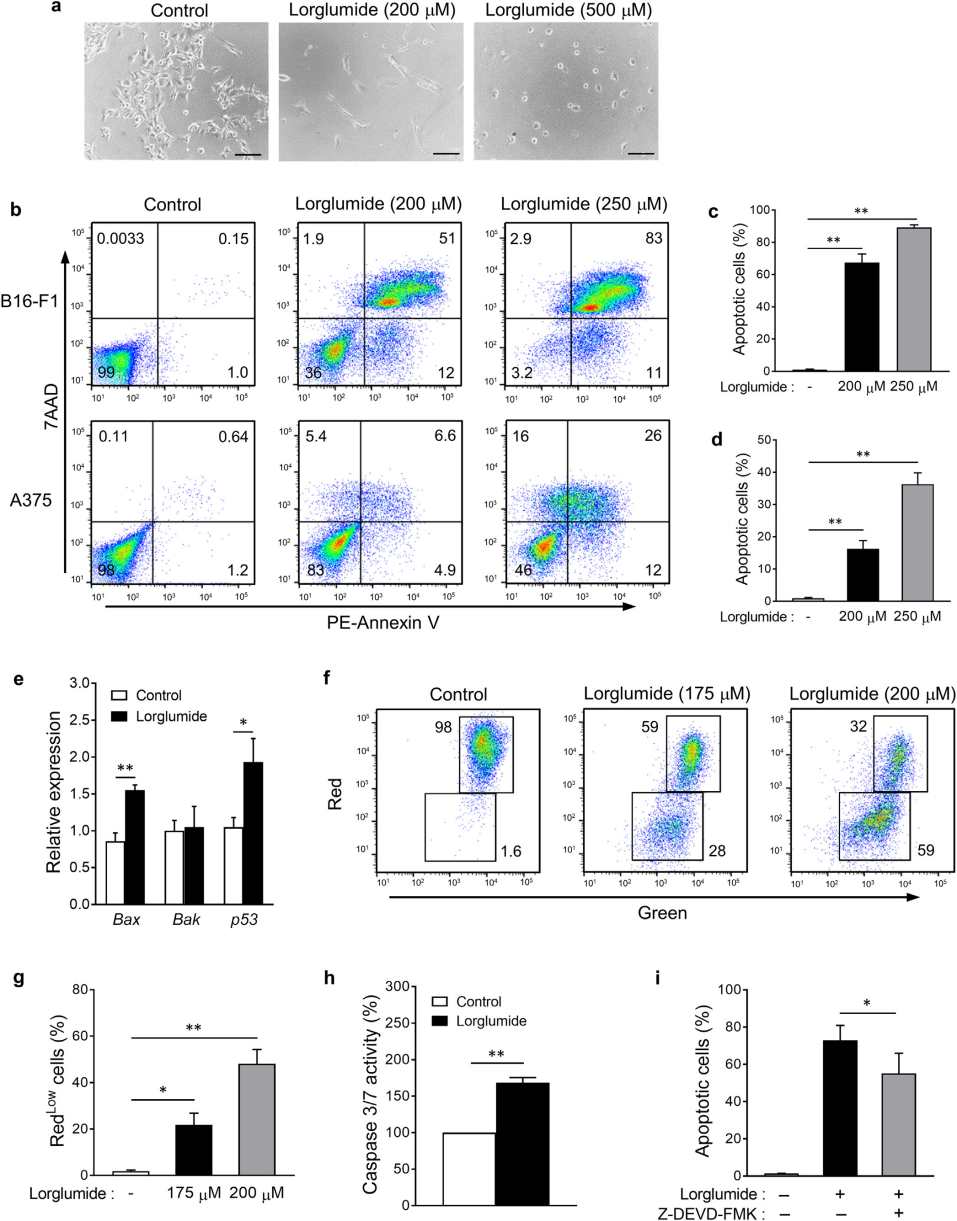
**CCK receptor antagonist induces the apoptosis of melanoma cells.** (**a**) Microscopic images of B16-F1 melanoma cells treated with lorglumide for 48 hours. Bar = 100 μm. (**b–d**) Apoptosis of B16-F1 and A375 melanoma cells was analyzed by staining with 7AAD and PE-Annexin V after the treatment with lorglumide for 48 hours. Percentages of apoptotic cells (7AAD^‒^Annexin V^+^ + 7AAD^+^Annexin V^+^) are shown as the mean ± SEM of three independent experiments in (**c)** B16-F1 and (**d)** A375 cells. ∗∗*P* < 0.01. (**e**) Real-time qPCR analysis of apoptosis regulatory genes in B16-F1 cells treated with lorglumide for 24 hours. Data are expressed as the mean ± SEM of three independent experiments performed in duplicate. ∗*P* < 0.05 and ∗∗*P* < 0.01. (**f**) The loss of mitochondrial membrane potential was detected using flow cytometry. Red^high^ and Red^low^ cells indicate cells with intact and disrupted mitochondrial membrane potential, respectively. The percentages of Red^low^ cells are shown as the mean ± SEM of three independent experiments in **g**. ∗*P* < 0.05 and ∗∗*P* < 0.01. (**h**) The activity of caspase-3/7 was determined using the Caspase-Glo 3/7 assay system and expressed as the mean ± SEM of three independent experiments performed in duplicate relative to the luminescence of the control. ∗∗*P* < 0.01. (**i**) B16-F1 cells were pretreated with Z-DEVD-FMK for 2 hours, followed by treatment with lorglumide for 48 hours. The percentages of apoptotic cells were determined using flow cytometry. Data are expressed as the mean ± SEM of three independent experiments. ∗*P* < 0.05. (**a, b, f**) Images are representative of three independent experiments. *P*-values were calculated using one-way ANOVA with Bonferroni’s adhoc test for multiple pairwise comparisons for **c**, **d, g,** and **i** and two-tailed *t*-test for **e** and **h**. 7AAD, 7-amino-actinomycin D; CCK, cholecystokinin; PE, phycoerythrin.

Morphological images of cultured melanoma cells were taken under a phase-contrast microscope. Whereas untreated B16-F1 cells grew well to form a monolayer, cells treated with lorglumide displayed an irregularly shrunken and rounded morphology (Figure 6a). These changes are thought to be induced during apoptosis (Elmore, 2007). Thus, we performed a flow cytometric analysis to detect phosphatidylserine exposure on the outer leaflets of the plasma membranes, which is a characteristic of apoptotic cells (Elmore, 2007). B16-F1 and A375 melanoma cells were treated with lorglumide for 48 hours and stained with 7-amino-actinomycin D (7AAD) and phycoerythrin-labeled Annexin V, which specifically binds to phosphatidylserine. As depicted in Figure 6b (upper panel), the treatment of B16-F1 cells with lorglumide increased the frequency of 7AAD^‒^/Annexin V^+^ early apoptotic cells and 7AAD^+^/Annexin V^+^ late apoptotic cells. Lorglumide increased the total percentage of early and late apoptotic B16-F1 cells in a dose-dependent manner (Figure 6c). The treatment of A375 human melanoma cells with lorglumide also significantly increased the number of apoptotic cells (Figure 6b [lower panel] and d).

### CCK receptor antagonist‒induced apoptosis is mitochondria dependent

To elucidate the mechanisms underlying the apoptosis induced by CCK receptor antagonists, we evaluated the expression levels of several apoptosis-related genes. The mRNA expression levels of *Bax*, *Bak*, and *p53* were measured using real-time qPCR in B16-F1 cells treated with lorglumide. The mRNA expression levels of the proapoptotic proteins *Bax* and *p53* but not *Bak* were significantly increased by lorglumide treatment (Figure 6e).

There are two pathways that transduce apoptotic signals: the death receptor‒mediated and mitochondrial pathways (Elmore, 2007). The death receptor‒mediated pathway is triggered by the binding of certain death receptor ligands to their cognate death receptors, such as the TNF receptor and Fas (Elmore, 2007). In contrast, DNA damage, chemotherapeutic agents, and UV irradiation induce apoptosis through the mitochondrial pathway, in which apoptotic stimuli decrease mitochondrial membrane potential, thereby inducing the release of apoptogenic factors from the mitochondria, leading to the activation of effector caspases, such as caspase-3, caspase-6, and caspase-7 (Czabotar et al., 2014; Elmore, 2007; Shalini et al., 2015). Because the proapoptotic protein BAX is involved in the regulation of mitochondrial membrane potential (Czabotar et al., 2014), the increased BAX expression in lorglumide-treated cells suggests that lorglumide induces apoptosis through the mitochondrial pathway.

To confirm this notion, we examined whether lorglumide affected the mitochondrial membrane potential. B16-F1 cells treated with lorglumide were stained with JC-1 fluorescent dye and analyzed using flow cytometry. JC-1 exerts strong red fluorescence in cells with intact mitochondrial membrane potential, whereas red fluorescence is decreased in cells with disrupted mitochondrial membrane potential. Most of the untreated control cells appeared as a Red^high^ population with intact mitochondrial membrane potential (Figure 6f and g). When cells were treated with lorglumide for 48 hours, the frequency of Red^low^ cells increased in a dose-dependent manner, indicating that lorglumide decreased the mitochondrial membrane potential.

We determined the enzymatic activity of effector caspases and found that the activity of caspase 3/7 in lorglumide-treated cells was 1.5 times higher than that in the untreated control cells (Figure 6h). To clarify the caspase-3 dependency of lorglumide-induced apoptosis, cells were treated with Z-DEVD-FMK, a specific inhibitor of caspase-3, before the induction of apoptosis by lorglumide. Flow cytometric analysis of apoptotic cells revealed that pretreatment of B16-F1 cells with Z-DEVD-FMK significantly reduced the number of apoptotic cells induced by lorglumide (Figure 6i), suggesting that the apoptosis of B16-F1 cells induced by the CCK receptor antagonist was at least partly dependent on caspase-3.

### CCK receptor antagonist inhibits the growth of melanoma and SCC in vivo

**Figure 7:**
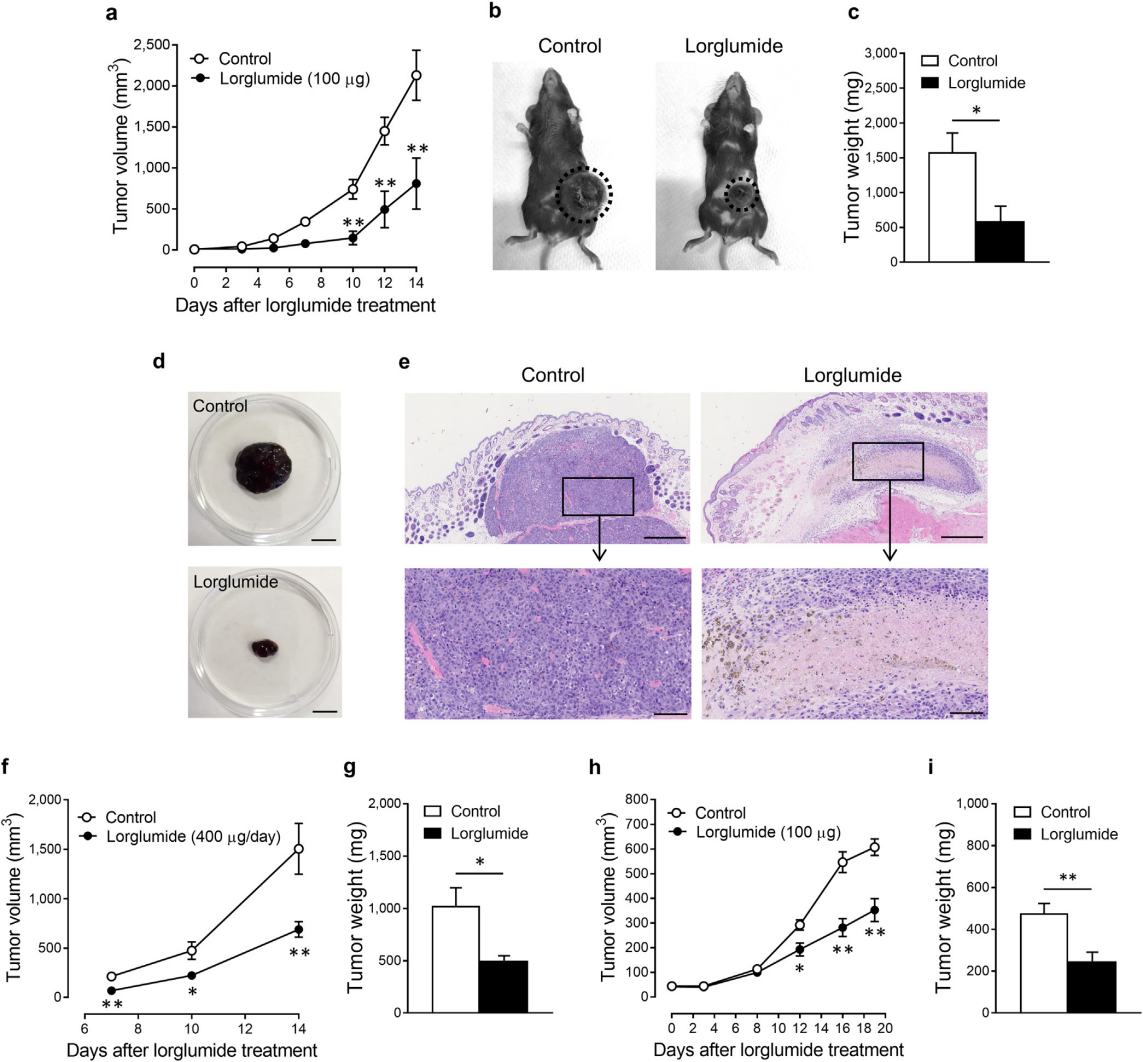
**CCK receptor antagonist inhibits the growth of melanoma and SCC in vivo.** (**a–g**) C57BL/6 mice implanted with B16-F1 melanoma cells received (**a–e**) intratumoral injections of lorglumide every 3 or 4 days or (**f, g**) epicutanous treatment with lorglumide five times a week. (**h, i**) *Scid/Scid* mice implanted with A431 cells received intratumoral injections of lorglumide every 2 or 3 days. (**a, f, h**) Tumor volume was determined, as described in the Materials and Methods section, and expressed as the mean ± SEM of two independent experiments performed with seven mice per group of each experiment. Tumor weight on (**c, g**) day 14 or (**i**) day 19 from the initial treatment with lorglumide was expressed as the mean ± SEM of two independent experiments performed with seven mice per group of each experiment. ∗*P* < 0.05 and ∗∗*P* < 0.01 versus control. Representative images of (**b**) transplanted mice and (**d**) tumor mass were taken on day 14, and (**e**) H&E staining was taken on day 6. Bar = 1 cm for **d** and 500 μm for **e (**upper panel) and 100 μm (lower panel). *P*-values were calculated using two-way ANOVA with Bonferroni’s adhoc test for multiple pairwise comparisons for **a, f,** and **h** and two-tailed *t*-test for **c, g,** and **i**. CCK, cholecystokinin; SCC, squamous cell carcinoma.

Finally, we evaluated the suppressive effect of the CCK receptor antagonist on the growth of skin tumors in vivo. Mice were subcutaneously implanted with B16-F1 melanoma cells, after which they received intratumoral injections of lorglumide every 3 or 4 days. By monitoring the tumor volume using a micrometer, we found that lorglumide significantly suppressed the increase in tumor volume when injected directly into the tumor (Figure 7a). On day 14, tumors were harvested from mice to measure their weight (Figure 7b–d). Intratumoral injection of lorglumide significantly reduced the tumor weight to approximately 38% of that of the saline-injected control tumors (Figure 7c). Histological examination revealed that some melanoma cells underwent nuclear division in the saline-injected tumor but not in the lorglumide-injected tumor, with necrosis mainly occurring in the lorglumide-injected tumor (Figure 7e). In the saline-injected specimens, small vessels were present around the tumor nests, whereas these vessels disappeared in the lorglumide-injected specimens (Figure 7e, upper panel). Notably, epicutaneous application of lorglumide significantly inhibited the tumor growth, as assessed by the tumor volume and weight (Figure 7f and g).

We further evaluated the in vivo antitumor effects of lorglumide in SCC. We used a xenograft model in which *Scid/Scid* mice were subcutaneously implanted with A431 SCC cells and received an intratumoral injection of lorglumide every 2 or 3 days. Lorglumide significantly suppressed the growth of SCC because the tumor volume and weight in the lorglumide-injected mice on day 19 were 58 and 52% of those in the control mice, respectively (Figure 7h and i).

## Discussion

Our study provides two important insights regarding the functions of CCKAR in skin tumors. First, melanoma and SCC cells pathologically employ CCK–CCK receptor engagement for tumor growth. Second, CCKAR antagonists may be potential therapeutic agents for the treatment of these tumors. Immunohistochemical staining showed that CCKAR was expressed in clinical skin specimens from human melanoma. Flow cytometric analysis revealed that CCKAR but not CCKBR was present in the melanoma cell lines (B16-F1 and A375) and A431 SCC cells, whereas HSC-1 SCC cells expressed both CCKAR and CCKBR. RT-PCR analysis showed that melanoma and SCC cell lines expressed the *CCK* mRNA. Many studies have shown the expression of CCK receptors and their ligands in various types of tumor cells, showing that CCK receptor signaling is involved in the regulation of tumor growth (Morimoto et al., 1993; Ponnusamy et al., 2016; Smith et al., 2016). Therefore, we investigated whether the blockade of CCK receptors with their antagonists affected the viability of melanoma and SCC cells. Results of the water-soluble tetrazolium salt-8 assay indicated that lorglumide, a CCKAR antagonist, decreased the viability of melanoma and SCC cells. CCK receptor antagonists decreased the viability of SCC cells through CCKAR but not through CCKBR. Using CFSE-labeled B16-F1 cells, we found that lorglumide suppressed cell division in melanoma cells. The detection of phosphatidylserine on the outer leaflet of the cell membrane further confirmed lorglumide-induced apoptosis in melanoma cell lines.

These results revealed that CCK promotes the proliferation and survival of melanoma and SCC cells through CCKAR in an autocrine or paracrine manner, and blockade of the CCK/CCKAR signaling suppresses their proliferation and survival. CCK is present in epidermal KCs (Funakoshi et al., 2019); hence, CCK derived from these cells may contribute to the growth of skin tumors. CCKAR antagonists may also function as inverse agonists, which directly induce apoptosis rather than block the antiapoptotic effects of endogenous CCK. CCK receptor antagonists suppress the proliferation of colon cancer cells even in the absence of exogenous or endogenous ligands, suggesting that the antiproliferative effect of CCK receptor antagonists does not merely stem from the blockade of the autocrine loop of the CCK receptor and its ligand (Forgue-lafitte et al., 1996).

Real-time qPCR analysis revealed that the expression of mRNA encoding the proapoptotic BCL-2 family protein, BAX, and tumor suppressor p53 increased after treatment with lorglumide. Activation of BAX disrupts the mitochondrial membrane potential, leading to the release of apoptogenic factors from the mitochondria, thereby activating the effector caspases (Czabotar et al., 2014). The mitochondrial membrane potential decreased in lorglumide-treated B16-F1 cells. Because p53 can directly activate BAX (Chipuk et al., 2004), lorglumide may induce apoptosis in melanoma cells through the mitochondrial pathway triggered by p53-dependent BAX activation. We found that the enzymatic activity of caspase-3/7 increased in lorglumide-treated B16-F1 melanoma cells. Because lorglumide-induced apoptosis was partially inhibited by the caspase-3 inhibitor, the CCK receptor antagonist induced apoptosis of melanoma cells, at least partly through caspase-3‒dependent mechanisms. The death receptor‒mediated pathway involves the activation of caspase-8, which subsequently leads to caspase-3 activation (Shalini et al., 2015). We determined the enzymatic activity of caspase-8 after treating B16-F1 cells with lorglumide and found no significant increase in caspase-8 activity (data not shown). Therefore, caspase-8 may not contribute to the activation of caspase-3 during lorglumide-induced apoptosis.

In a mouse model of melanoma and SCC, the intratumoral injection of lorglumide attenuated the growth of melanoma and SCC in vivo. Histological examination of the tumor specimen showed that lorglumide suppressed the cell division and promoted cell death, similar to the results of the in vitro studies. In addition to its direct effect on tumor cells, lorglumide may exert an antitumor effect in melanoma by suppressing angiogenesis, as reported in a human glioma xenograft model (Lefranc et al., 2004). Lorglumide exhibits an antitumor effect when applied externally to melanoma. Several studies have shown that the systemic administration of CCK receptor antagonists suppresses the growth of cancers, such as pancreatic and colon cancers (Beauchamp et al., 1985; Morimoto et al., 1993). However, in our mouse model of melanoma, oral administration of lorglumide failed to suppress melanoma growth (data not shown). This may be due to the difficulty in sufficiently increasing the intratumoral concentration of lorglumide to induce the apoptosis of melanoma cells when administered systemically.

**Figure 8:**
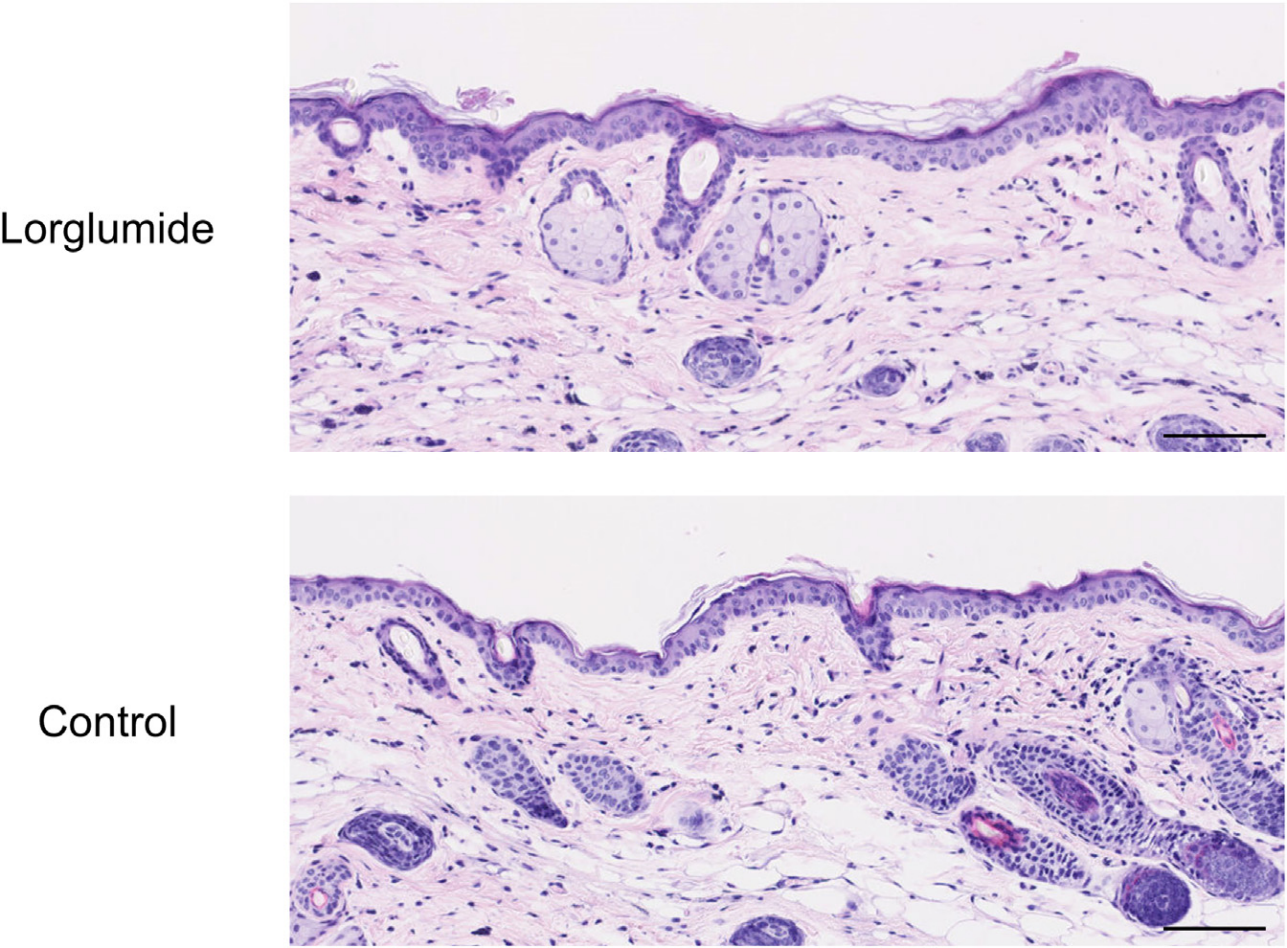
**H&E staining images of mouse skin painted with lorglumide.** Lorglumide dissolved in aqueous cream (400 μg/mouse) or aqueous cream (control) was applied epicutaneously onto the skin of the left frank five times a week. Twelve days later, the skin was excised and stained with H&E. Images are representative of two independent experiments (two mice per group). Bar = 100 μm.

Because CCK functions as a peptide hormone in digestive organs and the CNS, systemic treatment with a CCK receptor antagonist may induce broad unfavorable effects in these organs. In a therapeutic trial of chronic pancreatitis, oral administration of a CCKAR antagonist induced symptoms, such as abdominal fullness, diarrhea, and heartburn, in patients, although they were mild or moderate (Shiratori et al., 2002). We observed no significant histopathological changes in the epidermis when 400 μg of lorglumide was applied five times a week for 12 days (Figure 8). Therefore, local administration may be more appropriate than systemic administration of CCK receptor antagonists for the treatment of melanoma.

In conclusion, we showed that CCK receptor antagonists inhibit the proliferation and survival of melanoma and SCC cells in vitro and attenuate tumor growth in vivo by local treatment. Our results suggest the therapeutic potential of CCK receptor antagonists in the treatment of various types of skin cancer.

## Materials and Methods

### Clinical samples

Melanoma and nevi specimens were obtained from patients who had undergone surgery or biopsy at the Hamamatsu University School of Medicine (Hamamatsu, Japan). The experimental protocol for the human study was conducted in accordance with the principles of the Declaration of Helsinki and was approved by the medical ethical committees of Hamamatsu University School of Medicine (number 19-262). Written informed consent was obtained from the patients, and if not, the opt-outs for the protocol were also opened to the general public online at Hamamatsu University School of Medicine.

### Immunohistochemistry

Melanoma and nevi specimens were fixed in 10% buffered formalin and embedded in paraffin. Sections were deparaffinized and stained with H&E. For immunohistochemistry, deparaffinized sections were stained with the following primary antibodies for 1 hour at room temperature: anti-CCKAR polyclonal (PA3-116, 1:350; Thermo Fisher Scientific, Waltham, MA), anti-CCK polyclonal (2 μg/ml; ab27441, 1:100; Abcam, Cambridge, United Kingdom), rabbit IgG isotype control (2 μg/ml; ab37415, 1:250; Abcam), anti‒HMB-45 monoclonal (0.5 μg/ml; M0634, 1:50; Agilent Technologies, Santa Clara, CA), anti-Melan A monoclonal (2 μg/ml; M7196, 1:50; Agilent Technologies), and mouse IgG1 isotype control monoclonal (2 μg/ml; ab91353, 1:50; Abcam) antibodies. Immunoreactivity was detected using Histofine Simple Stain Max PO (Nichirei Biosciences, Tokyo, Japan). The color was developed using 3-amino-9-ethylcarbazole as the substrate.

### Cell lines

B16-F1 mouse melanoma and A375 human melanoma cells were maintained in DMEM supplemented with 10% fetal bovine serum. A431 and HSC-1 human SCC cells were grown in RPMI-1640 medium supplemented with 10% fetal bovine serum and 1% nonessential amino acids (FUJIFILM Wako Chemical, Miyazaki, Japan) and DMEM supplemented with 20% fetal bovine serum, respectively. Jurkat cells were grown in RPMI-1640 medium supplemented with 10% fetal bovine serum. Cells were passaged every 3 days (B16-F1, A375, and Jurkat cells) or 4 days (A431 and HSC-1 cells) at subconfluent cell densities.

### Flow cytometric analysis of CCK receptors

To analyze CCK receptor expression, 10^6^ cells were stained with 1 μg of the anti-CCKAR (bs-11514R; Bioss Antibodies, Woburn, MA), anti-CCKBR (ACR-042; Alamone Labs, Jerusalem, Israel), or rabbit IgG isotype control (ab37415; Abcam) antibody for 1 hour on ice, followed by incubation with Alexa Fluor 647‒conjugated anti-rabbit IgG (460414; BioLegend, San Diego, CA) for 30 minutes on ice. After incubation with 7AAD (BD Biosciences, San Diego, CA) for 10 minutes, the expression levels of CCK receptors in 7AAD^‒^ live cells were determined using FACScantoII (BD Biosciences). Data were analyzed using the FlowJo software (BD Biosciences).

### RT-PCR analysis of CCK

Total RNA from melanoma cells, SCC cells, and normal human epidermal KCs (Kurabo, Osaka, Japan) was purified using Isogen II (Nippon Gene, Tokyo, Japan) and reverse transcribed using the PrimeScript RT reagent Kit with gDNA Eraser (Takara Bio, Shiga, Japan). Mouse skin RNA was purified using the RNeasy Fibrous Tissue Mini Kit (Qiagen, Hilden, Germany) and transcribed as a positive control for mouse CCK. *CCK* and *β-actin* genes were amplified using a T100 Thermal Cycler (Bio-Rad Laboratory, Hercules, CA) with Ex Taq DNA polymerase (Takara Bio) and a pair of specific primers as follows: 5′-GGTCCGCAAAGCTCCTTCT-3′ forward for mouse *CCK* and 5′-AGACATTAGAGGCGAGGGGT-3′ reverse for mouse *CCK,* 5′-GGGTATCGCAGAGAACGGAT-3′ forward for human *CCK* and 5′-GTGTGGTTGCACTGGACAATC-3′ reverse for human *CCK,* 5′-AGTGTGACGTTGACATCCGT-3′ forward for mouse *β-actin* and 5′-GAGTACTTGCGCTCAGGAGG-3′ reverse for mouse *β-actin*, and 5′-CTTCTACAATGAGCTGCGTG-3′ forward for human *β-actin* and 5′-TCATGAGGTAGTCAGTCAGG-3′ reverse for human *β-actin*.

### Water-soluble tetrazolium salt-8 assay

Cells were seeded into each well of a 96-well plate (B16-F1 and A375 cells, 2 × 10^3^ cells per well; A431 and HSC-1 cells, 4 × 10^3^ cells per well). After overnight culture at 37 °C, the cells were treated with lorglumide (Abcam), proglumide (Sigma-Aldrich, St. Louis, MO), or loxiglumide (Sigma-Aldrich) for 48 hours. A total of 10 μl of water-soluble tetrazolium salt-8 (Dojindo Molecular Technologies, Kumamoto, Japan) was added to each well of a 96-well plate, and the cells were incubated for another 3 hours at 37 °C. Optical density was measured using an iMark Microplate Absorbance Reader (Bio-Rad Laboratory) at 450 nm.

### Proliferation assay

B16-F1 cells were labeled with CFSE using the CFSE Cell Division Assay Kit (Cayman Chemical, Ann Arbor, MI), according to the manufacturer’s instructions. The cells were then cultured in a six-well plate (5 × 10^5^ cells per well) for 4–5 hours to facilitate adherence to the plates, followed by treatment with lorglumide. After 48 hours, the fluorescence intensity of CFSE was determined using FACScanto II.

### Cell cycle analysis

B16-F1 cells were seeded in a six-well plate (2 × 10^5^ cells per well) and cultured overnight at 37 °C. The cells were treated with lorglumide for 48 hours. After treatment with lorglumide, cells were fixed with 70% ethanol and incubated at –20 °C for 2 hours. The cells were stained with propidium iodide/RNase Staining Buffer (BD Biosciences) for 15 minutes, and the fluorescence intensity of propidium iodide was determined using FACScantoII. Cell cycle analysis was performed using the FlowJo software.

### Apoptotic and mitochondrial membrane potential assays

The phycoerythrin-Annexin V Apoptosis Detection Kit 1 (BD Biosciences) was used to detect the apoptotic cells. Cells were treated with lorglumide in a six-well plate (2 × 10^5^ cells per well), as described earlier. In some experiments, cells were pretreated with the caspase-3 inhibitor, Z-DEVD-FMK (40 μM; BD Biosciences), for 2 hours before treatment with lorglumide. After treatment with lorglumide, the cells were stained with phycoerythrin-labeled Annexin V and 7AAD, according to the manufacturer’s instructions. Because trypsinization may damage the cell membrane and expose phosphatidylserine outside the membrane, cells that adhered to the culture plates were stained with phycoerythrin-labeled Annexin V and 7AAD and harvested through trypsinization. To measure the mitochondrial membrane potential, cells were stained with MitoPT JC-1 (ImmunoChemistry Technologies, Bloomington, MN) for 30 minutes at 37 °C. The stained cells were then analyzed using FACScanto II.

### Real-time qPCR analysis

Real-time qPCR analysis was performed to compare the levels of gene expression using the Thermal Cycler Dice Real Time System II (Takara Bio). Expression levels were normalized to *GAPDH* mRNA, which was amplified using a ROX-labeled probe (5′-ACGACCCCTTCATTGACCTCAACTACATGG-3′) and a pair of specific primers (forward 5′-GTCGGTGTGAACGGATTTGG-3′ and reverse 5′-TTTGCCGTGAGTGGAGTCAT-3′). TaqMan probes obtained from Thermo Fisher Scientific were as follows: mouse *Bax*, Mm00432051_m1; mouse *Bak*, Mm00432045_m1; and mouse *p53*, Mm01731290_g1.

### Measurement of caspase activity

Caspase activity was measured using the Caspase-Glo 3/7 Assay System (Promega, Madison, WI). B16-F1 cells were treated with lorglumide for 48 hours and Caspase-Glo reagent was added at the end of the culture. The plate was incubated for 2 hours, and luminescence was measured using a Synergy H1 microplate reader (BioTek, Winooski, VT).

### Mice and tumor models

Female C57BL/6 (Japan SLC, Shizuoka, Japan) and C.B-17/Icr-*Scid/Scid* mice (CLEA Japan, Tokyo, Japan) were subcutaneously injected with 10^5^ B16-F1 cells and 5 × 10^6^ A431 cells, respectively, into the shaved left flank. When the tumor was palpable, the transplanted mice were randomly divided into two groups. C57BL/6 and C.B-17/Icr-*Scid/Scid* mice received intratumoral injections of lorglumide (100 μg; Abcam) or saline every 3–4 or 2–3 days, respectively. For epicutaneous treatment of melanoma with lorglumide, lorglumide dissolved in aqueous cream (400 μg/mouse) was applied epicutaneously onto the tumor mass five times a week. Tumor size was measured using a micrometer (Mitutoyo, Kanagawa, Japan), and tumor volume was calculated as follows: length × width^2^ × 0.5. The experiments were performed according to the guidelines of Hamamatsu University School of Medicine.

### Statistical analysis

All statistical analyses were performed using Statistical Package for the Social Sciences, version 25 (IBM, Armonk, NY). The distribution of data was analyzed using the Shapiro–Wilk normality test and quantile‒quantile plots. To determine statistical significance, ANOVA was performed, followed by Bonferroni’s adhoc test for multiple pairwise comparisons. Two-tailed Student’s *t*-test was used to compare the means between two groups. The level of significance was set at *P* < 0.05.

### Data availability statement

No datasets were generated or analyzed during this study.

## ORCIDs

Atsuko Funakoshi: http://orcid.org/0000-0003-0383-8901

Tetsuya Honda: http://orcid.org/0000-0003-2355-4869

Taisukke Ito: http://orcid.org/0000-0002-9274-7050

Yoshiki Tokura: http://orcid.org/0000-0001-7452-6919

## Conflict of Interest

The authors state no conflict of interest.
